# Academ Virus, a Novel Hantavirus in the Siberian Mole (*Talpa altaica*) from Russia

**DOI:** 10.3390/v14020309

**Published:** 2022-02-02

**Authors:** Liudmila N. Yashina, Victor V. Panov, Sergey A. Abramov, Natalia A. Smetannikova, Ekaterina M. Luchnikova, Tamara A. Dupal, Anton V. Krivopalov, Satoru Arai, Richard Yanagihara

**Affiliations:** 1 State Research Center of Virology and Biotechnology VECTOR, 630559 Koltsovo, Russia; smetannikova@vector.nsc.ru; 2 Institute of Systematics and Ecology of Animals, 630091 Novosibirsk, Russia; panovv53@mail.ru (V.V.P.); terio@eco.nsc.ru (S.A.A.); dupalgf54@gmail.com (T.A.D.); krivopalov@gmail.com (A.V.K.); 3 Department of Ecology and Environmental Management, Institute of Biology, Ecology and Natural Resources, Kemerovo State University, 650099 Kemerovo, Russia; lut@yandex.ru; 4 Center for Surveillance, Immunization, and Epidemiologic Research, National Institute of Infectious Diseases, Tokyo 162-8640, Japan; arais@nih.go.jp; 5 John A. Burns School of Medicine, University of Hawaii at Manoa, Honolulu, HI 96813, USA

**Keywords:** *Hantaviridae*, hantavirus, mole, evolution, Russia

## Abstract

To date, six hantavirus species have been detected in moles (family Talpidae). In this report, we describe Academ virus (ACDV), a novel hantavirus harbored by the Siberian mole (*Talpa altaica*) in Western Siberia. Genetic analysis of the complete S-, M-, and partial L-genomic segments showed that ACDV shared a common evolutionary origin with Bruges virus, previously identified in the European mole (*Talpa europaea*), and is distantly related to other mole-borne hantaviruses. Co-evolution and local adaptation of genetic variants of hantaviruses and their hosts, with possible reassortment events, might have shaped the evolutionary history of ACDV.

## 1. Introduction

Recently, the host range of hantaviruses (family *Hantaviridae*) has been expanded with the discovery of genetically distinct hantaviruses in multiple species of shrews, moles, and bats [1,2]. Although several rodent-borne hantaviruses are known to cause hemorrhagic fever with renal syndrome or hantavirus cardiopulmonary syndrome [3,4], the pathogenicity of non-rodent-associated hantaviruses remains unclear, despite the infrequent detection of antibodies against certain shrew-borne hantaviruses in humans [5,6]. The genome of hantaviruses comprises three negative-polarity RNA segments: small (S), medium (M), and large (L), encoding a nucleocapsid (N) and occasionally nonstructural (NSs) protein, envelope glycoproteins (Gn and Gc), and an RNA-dependent RNA polymerase (RdRP), respectively. Currently, mammalian-associated hantaviruses are classified into four genera: *Orthohantavirus*, *Loanvirus*, *Mobatvirus*, and *Thottimvirus* [7].

With the discovery of highly divergent hantaviruses in shrews, moles, and bats [1,2], long-held hypotheses of the co-evolution of rodent-borne hantaviruses and their hosts have changed to concepts of a far more complex evolutionary history, with cross-species transmission and host switching [8,9,10,11,12,13] and reassortment events [14,15,16,17,18]. Genetically distinct hantaviruses have been identified in five species of moles (family Talpidae) from Eurasia and North America and mole-borne hantaviruses demonstrate distinct evolutionary histories [9,17,19,20,21,22]. The first mole-borne hantavirus, Nova virus (NVAV) [21], is widespread across the geographic distribution of its host, the European mole (*Talpa europaea*) [23]. Phylogenetic analysis of the full-length segments showed that NVAV was most closely related to hantaviruses harbored by bats and classified in the *Mobatvirus* genus.

Recently, NVAV and Bruges virus (BRGV) were found co-circulating among European moles in Belgium, Germany, and the United Kingdom [17]. Phylogenetic placement of one of these viruses (BRGV, member of *Orthohantavirus* genus) corresponded to the co-evolution hypothesis, while the position of the second hantavirus (NVAV, member of *Mobatvirus* genus) suggested cross-species transmission and an ancient reassortment event. Parallel evolution associated with cross-species transmission had been shown for other orthohantaviruses harbored by shrew moles. Phylogenetic analysis and host–parasite evolutionary comparisons showed that Asama virus (ASAV) from the Japanese shrew mole (*Urotrichus talpoides*) and Oxbow virus (OXBV) from the American shrew mole (*Neurotrichus gibbsii*) were related to soricine shrew-borne hantaviruses from Eurasia and North America, respectively [9,20].

Host-switching events have been suggested between mole-borne and rodent-borne hantaviruses. Rockport virus (RKPV) identified in the eastern mole (*Scalopus aquaticus*) shared a more recent common ancestor with cricetid-rodent-borne hantaviruses, which are sympatric across the eastern United States [22]. Dahonggou Creek virus (DHCV), harbored by the long-tailed mole (*Scaptonyx fusicaudus*), is a member of the *Thottimvirus* genus, joining crocidurine and myosoricine shrew-borne hantaviruses [19]. Further investigation of hantavirus–reservoir relationships among moles is important for understanding hantavirus evolution. Here, we report the detection, genomic characterization, and geographic distribution of a new hantavirus, named Academ virus (ACDV), in the Siberian mole (*Talpa altaica*).

## 2. Materials and Methods

### 2.1. Trapping and Sample Collection

During June to August 2017–2021, Siberian moles were trapped in Western Siberia, Russia. All wildlife field operations, including the responsible treatment of animals, met the guideline requirements of the order of the Russian High and Middle Education Ministry (No. 742 issued on 13 November 1984) and by the Federal Law of the Russian Federation (No. 498-FZ issued on 19 December 2018). Collection sites in the Novosibirsk Oblast were located near Academgorodok town, separate district of Novosibirsk City (54.82484° N/83.09392° E), Teletskoye Lake (51.79424° N/87.30447° E) in the Altai Republic, and around the settlement Azhendarovo (54.76237° N/87.03094° E and 54.74537° N/87.02093° E) in the Kemerovo Oblast. Permanent pitfall traps were used to capture moles near Academgorodok and around Azhendarovo. Lethal spring-loaded scissor traps, placed in burrows, were used at Teletskoye Lake. Lung samples were collected aseptically and stored in RNAlater™ (Qiagen, Hilden, Germany) before analysis.

### 2.2. RNA Extraction and RT-PCR Analysis

Total RNA was extracted from lung tissues, using the RNeasy MiniKit (Qiagen, Hilden, Germany), then reverse transcribed, using the Expand reverse transcriptase (Roche, Basel, Switzerland) with universal oligonucleotide primer (OSM55, 5′–TAGTAGTAGACTCC–3′), designed from the conserved 3′ end of the S-, M-, and L-segments of hantaviruses. For initial screening by nested RT-PCR, previously described genus-specific oligonucleotide primers targeting the partial L-segment sequence were used [24]. Oligonucleotide primers specific for ACDV S and M-segments (Supplementary Table S1) were designed from consensus regions of other hantaviruses. Amplicons were separated by electrophoresis on 1.2% agarose gels and purified using the QIAQuick Gel Extraction Kit (Qiagen, Hilden, Germany). DNA was sequenced directly using an ABI Prism 310 Genetic Analyzer (Applied Biosystems, Foster City, CA, USA).

### 2.3. Genetic and Phylogenetic Analysis

Pairwise alignment and comparison of full-length coding regions of the S-, M-, and partial L-segment nucleotide and amino acid sequences of hantaviruses from *T. altaica*, captured in Russia, with representative rodent-, shrew-, mole-, and bat-borne hantaviruses were performed, using the ClustalW in BioEdit [25,26,27]. Phylogenetic trees were generated using the Markov chain Monte Carlo (MCMC) methods MrBayes 3.1.2 [28], under the best-fit general time-reversible model of nucleotide evolution with gamma-distributed rate heterogeneity and invariable sites (GTR + I + Г) [29]. The best-fit model was selected with jModelTest version 2.1.9 [30] for phylogenetic trees. Two replicate Bayesian Metropolis–Hastings MCMC runs, each consisting of six chains of 10 million generations sampled every 100 generations with a burn-in of 25,000 (25%), resulted in 150,000 trees overall.

## 3. Results

### 3.1. Genetic Analysis

**Figure 1:**
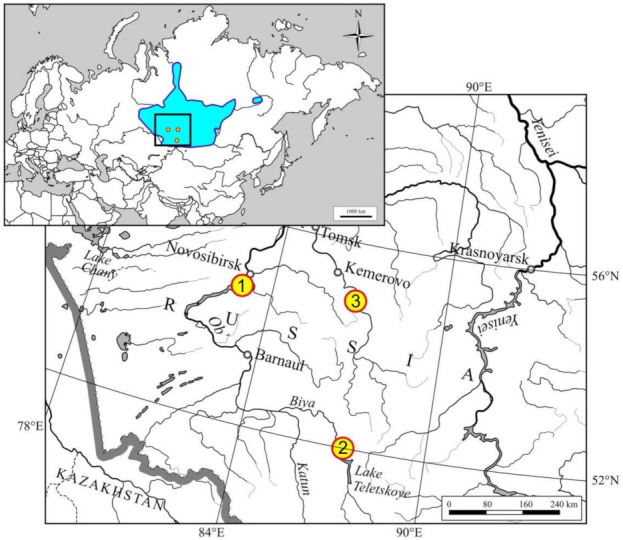
Map, showing the locations of the collection sites in West Siberia, Russia, where hantavirus-infected Siberian moles were captured. (1) Academgorodok, (2) Teletskoye, (3) Azhendarovo. The inset shows the geographic range of *Talpa altaica* (area colored in blue).

During 2017–2021, 18 Siberian moles were captured at three localities of Novosibirsk, Kemerovo Oblast, and Altai Republic, in Western Siberia (Table 1 and Figure 1). RNAlater™-preserved lung specimens were analyzed for hantavirus RNA by nested RT-PCR using oligonucleotide primers directed at the RdRP gene. Hantaviral RNA was detected in 14 of 18 Siberian moles (77.8%). The geographic distribution of positive samples included all three sampling sites. A new hantavirus was detected in one of two Siberian moles, captured in 2017 in the forest surrounding Academgorodok, a separate district of Novosibirsk City. The 346-nucleotide fragment of the L-segment displayed relatively low sequence similarity to other hantaviruses and was most closely related to BRGV. This new hantavirus was designated ACDV strain Academ-Ta450/Russia/2017 according to the capture site. Further analysis of five hantavirus-positive samples collected in 2019 and 2021 (Table 1) in the same site demonstrated close identity of partial L-segment sequences (98.0–99.7% nucleotide and 100% amino acid identity). By contrast, ACDV sequences recovered from six of seven Siberian moles captured in 2018 and 2020 near Teletskoye Lake, Altai Republic, demonstrated a higher level of sequence divergence between each other (0.3–11.8% nucleotide, 0–1.7% amino acid). Moreover, relatively diverse ACDV sequences were detected between two of four Siberian moles captured at the third locality, Azhendarovo in Kemerovo Oblast (14.9% nucleotide, 1.7% amino acid). Although ACDV strains from the three sites had nucleotide identities ranging from 80.7 to 84.2%, the corresponding amino acid sequences were highly conserved (96.5–99.1%).

**Figure 2:**
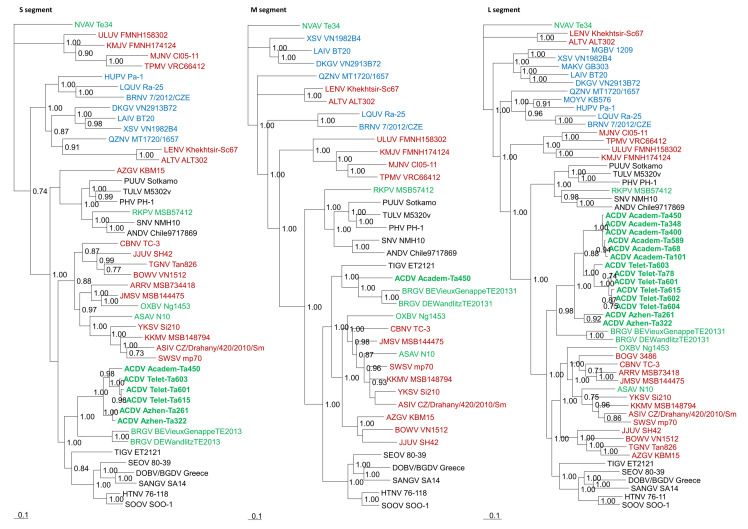
Phylogenetic trees generated by the Bayesian method, under the best-fit GTR+I+Γ model of evolution, based on the S-, M-, and L-genomic segments of ACDV (green, bold lettering). The phylogenetic positions of ACDV strains in Russia are shown in relationship to mole-borne hantaviruses (green): Asama orthohantavirus (ASAV N10, S: EU929072; M: EU929075; L: EU929078) from *Urotrichus talpoides*, Nova mobatvirus (NVAV Te34, S: KR072621, M: KR072622, L: KR072623), and Bruges orthohantavirus (BRGV BE/VieuxGenappeTE2013, S: KR072621; M: KR072622; L: KR072623 and BRGV DEWandlitzTE2013, S: MF683844; M: MF683845; L: MF683846) from *Talpa europaea*, Rockport orthohantavirus (RKPV MSB57412, S: HM015223; M: HM015222; L: HM015221) from *Scalopus aquaticus*, and Oxbow orthohantavirus (OXBV Ng1453, S: FJ5339166; M: FJ539167; L: FJ593497) from *Neurotrichus gibbsii*. Shrew-borne hantaviruses (red lettering) include Thottapalayam thottimvirus (TPMV VRC66412, S: AY526097, M: EU001329, L: EU001330) from *Suncus murinus*, Imjin thottimvirus (MJNV Cl05-11, S: EF641804; M: EF641798; L: EF641806) from *Crocidura lasiura*, Uluguru thottimvirus (ULUV FMNH158302, S: JX193695; M: JX193696; L: JX193697) from *Myosorex geata*, Kilimanjaro thottimvirus (KMJV FMNH174124, S: JX193698; M: JX193699; L: JX193700) from *Myosorex zinki*, Jeju orthohantavirus (JJUV SH42, S: HQ663933; M: HQ663934; L: HQ663935) from *Crocidura shantungensis*, Cao Bằng orthohantavirus (CBNV TC-3, S: EF543524; M: EF543526; L: EF543525) from *Anourosorex squamipes*, Azagny orthohantavirus (AZGV KBM15, S: JF276226; M: JF276227; L: JF276228) from *Crocidura obscurior*, Bowé orthohantavirus (BOWV VN1512, S: KC631782; M: KC631783; L: KC631784) from *Crocidura douceti*, prototype Seewis orthohantavirus (SWSV mp70, S: EF636024; M: EF636025; L: EF636026), and Altai virus (ALTV ALT302, S: MK340902; M: MK340903; L: MT648514) from *Sorex araneus*, Lena virus (LENV Khekhtsir-Sc67, S: MH499470; M: MH499471; L: MH499472) from *Sorex caecutiens*, Asikkala virus (ASIV CZ/Drahany/420/2010/Sm, S: KC880342; M: KC880345; L: KC880348) from *Sorex minutus*, Jemez Springs orthohantavirus (JMSV MSB144475, S: FJ593499; M: FJ593500; L: FJ593501) from *Sorex monticolus*, Tanganya virus (TGNV Tan826, S: EF050455; L: EF050454) from *Crocidura theresea*, Ash River virus (ARRV MSB73418, S: EF650086; L: EF619961) from *Sorex cinereus*, Yakeshi virus (YKSV Si-210, S: JX465423; M: JX465403; L: JX465389) from *Sorex isodon*, Kenkeme virus (KKMV MSB148794, S: GQ306148; M: GQ306149; L: GQ306150 and KKMV Fuyuan Sr326, S: NC_034559; M: KJ857337; L: KJ857320) from *Sorex roboratus*, and Boginia virus (BOGV 3486, L: KM394262) from *Neomys fodiens*. Also shown are representative rodent-borne hantaviruses (black lettering), including Sin Nombre orthohantavirus (SNV NMH10, S: NC_005216; M: NC_005215; L: NC_005217), Andes orthohantavirus (ANDV Chile9717869, S: AF291702; M: AF291703; L: AF291704), Prospect Hill orthohantavirus (PHV PH-1, S: Z49098; M: X55129; L: EF646763), Tula orthohantavirus (TULV M5302v, S: NC_005227; M: NC_005228; L: NC_005226), Puumala orthohantavirus (PUUV Sotkamo, S: NC_005224; M: NC_005223; L: NC_005225), Sangassou orthohantavirus (SANGV SA14, S: JQ082300; M: JQ082301; L: JQ082302), Soochong orthohantavirus (SOOV SOO-1, S: AY675349; M: AY675353; L: DQ056292), Dobrava/Belgrade orthohantavirus (DOBV/BGDV Greece, S: NC_005233; M: NC_005234; L: NC_005235), Hantaan orthohantavirus (HTNV 76-118, S: NC_005218; M: NC_005219; L: NC_005222), and Seoul orthohantavirus (SEOV 80-39, S: NC_005236; M: NC_005237; L: NC_005238), and Tigray virus (TIGV ET2121, S: KU934010; M: KU934009; L: KU934008) from *Stenocephalemys albipes*. Bat-borne hantaviruses (blue lettering) include Brno loanvirus (BRNV 7/2012/CZE, S: KX845678; M: KX845679; L: KX845680) from *Nyctalus noctula*, Láibīn mobatvirus (LAIV BT20, S: KM102247; M: KM102248; L: KM102249) from *Taphozous melanopogon*, Xuân Sơn mobatvirus (XSV VN1982B4, S: KC688335; L: JX912953) from *Hipposideros pomona*, Quezon mobatvirus (QZNV MT1720/1657, S: KU950713; M: KU950714; L: KU950715) from *Rousettus amplexicaudatus*, Mouyassué virus (MOYV KB576, L: JQ28771) from *Neoromicia nanus*, Makokou virus (MAKV GB303, L: KT316176) from *Hipposideros ruber*, Huángpí virus (HUPV Pa-1, S: JX473273, and L: JX465369) from *Pipistrellus abramus*, Lóngquán loanvirus (LQUV Ra-25, S: JX465415; M: JX465397; and L: JX465381) from *Rhinolophus sinicus*, Đakrông mobatvirus (DKGV VN2913B72, S: MG663536; M: MG663535; L: MG663534) from *Aselliscus stoliczkanus*, respectively. The numbers at each node are posterior node probabilities (>0.7), based on 150,000 trees: two replicate Markov Chain Monte Carlo runs consisting of six chains of 10 million generations each sampled every 100 generations with a burn-in of 25,000 (25%). The scale bar indicates the nucleotide substitutions per site.

The 1791-nucleotide S-segment of ACDV (prototype strain Academ-Ta450/Russia/2017) encoded an N protein of 430 amino acids in length. An additional open reading frame on the nonstructural NSs protein was not present as in other mole-borne hantaviruses. The intraspecies variability of the new virus was estimated based on complete S-segment coding sequences that were recovered from six hantavirus RNA-positive samples. These new strains showed divergent L-segment sequences and were found in three geographically distant sites (strains Academ-Ta450, Telet-Ta601, Telet-Ta603, Telet-Ta615, Azhen-Ta261, Azhen-Ta322). The coding sequences of the new strains were of the same length, minor insertions/deletions were observed in the noncoding 3′ termini. Comparative analysis demonstrated relatively high genetic diversity between new strains, 3.6–16.3% at the nucleotide level, while the amino acid sequences were conserved (divergence was less than 1.4%). Pairwise alignment and comparison of the ACDV S-segment with representative hantaviruses belonging to the four genera of the *Mammantavirinae* subfamily (Figure 2) showed considerable divergence, ranging between 31–47% and 26–53% at the nucleotide and amino acid level, respectively.

The complete 3648-nucleotide M-segment of ACDV (prototype strain Academ-Ta450/Russia/2017) contained a single ORF encoding the 1138-amino acid glycoprotein precursor (GP) of the Gn and Gc glycoproteins, separated by a WAVSA pentapeptide. The same motif was found in hantavirus ALTV (ALT302) and LENV (Khekhtsir-Sc67) from *Sorex* shrews in Russia and BRGV from the European mole (BE/Vieux-Genappe/TE/2013) in Belgium [17,31,32]. Analysis of the complete M-coding sequence revealed more than 29% nucleotide and amino acid sequence differences between ACDV and the most closely related BRGV virus (strains BE/Vieux-Genappe/TE/2013, DE/Wandlitz/TE/2013) and considerable divergence from other representative hantaviruses both at the nucleotide (>35%) and amino acid (>37%) levels. The observed amino acid pairwise evolutionary distance (PED) values ranged between 0.3 and 1.0, thus exceeding current species demarcation criteria of a PED cut-off value of 0.1 [7], suggesting that ACDV represents a new hantavirus species.

### 3.2. Phylogenetic Analysis

Phylogenetic trees, based on the coding regions of the full-length S-, M, and partial L-segments, were constructed by Bayesian methods. A phylogenetic tree of the S-segment demonstrated that ACDV from six Siberian moles had a common ancestry with BRGV from two European moles, segregated into separate clades with host species, and revealed the closest relationship to hantaviruses associated with hosts from the Muridae family (Figure 2). ACDV strains were divided into three sublineages with clear geographic clusters. Strains from Academgorodok were grouped into one sublineage, strains from Teletskoye formed a second sublineage, and strains from Azhendarovo were divided into another sublineage. A phylogenetic tree of the 14 partial L-segment sequences revealed similar topology. A phylogenetic tree of the complete coding sequence of the M-segment showed that ACDV and BRGV clustered closer to hantaviruses associated with hosts from the Soricidae family, suggesting possible reassortment events during the evolution of ACDV and BRGV.

**Figure 3:**
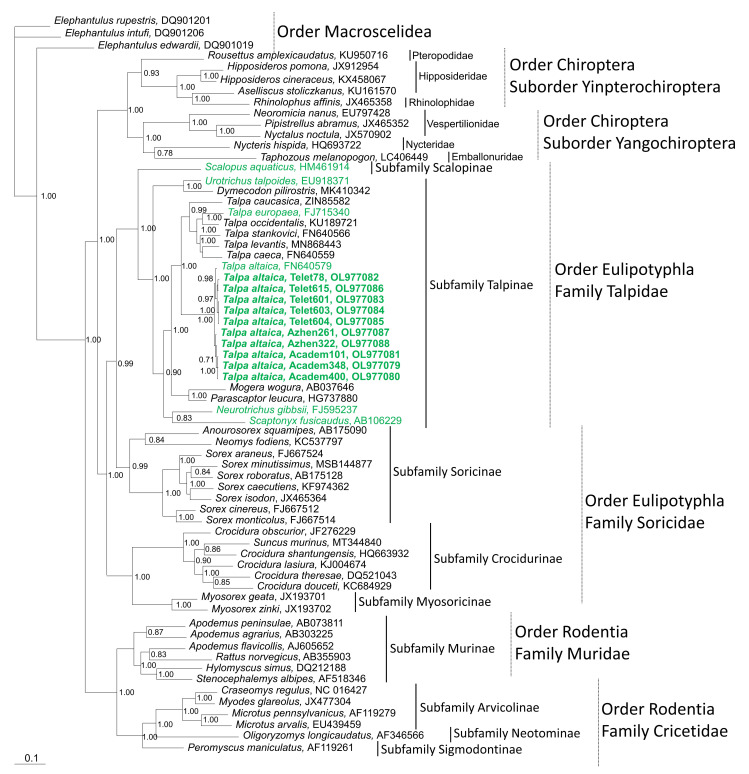
Bayesian phylogenetic tree based on 1140 nucleotides of the cytochrome b mtDNA of shrews and moles (order Eulipotyphla, families Talpidae and Soricidae), rodents (order Rodentia, families Muridae and Cricetidae), and bats (order Chiroptera, suborders Yinpterochiroptera and Yangochiroptera). The tree was rooted using *Elephantulus* (order Macroscelidea, GenBank Nos. DQ901019, DQ901206, and DQ901201) as the outgroup. FN640579 is from a Siberian mole captured in Teletskoye. *Talpa altaica* from this study are shown in bolded green lettering. Other mole species harboring hantaviruses are shown in unbolded green lettering. The number at each node indicates posterior probability values (>0.7), based on 15,000 trees: two replicate Markov chain Monte Carlo runs, consisting of six chains of one million generations each sampled every 100 generations with a burn-in of 2500 (25%). The scale bar indicates the number of nucleotide substitutions per site. GenBank accession numbers are shown for taxa.

Phylogenetic analysis of the cytochrome b (cyt B) gene from ACDV-positive moles confirmed the host identity as *Talpa altaica* (GenBank Accession Number OL977079–OL977088). Newly acquired host mtDNA sequences from Siberian moles segregated into distinct geographic-specific lineages (Figure 3). Sequences from Siberian moles, captured at Teletskoye and Azhendarovo, differentiated into three and two sublineages, respectively. Moles, captured at Academgorodok, had minimal cyt B sequence diversity and grouped into a single sublineage (Figure 3).

## 4. Discussion

Here, we describe a new hantavirus, named ACDV, in the Siberian mole, captured in Western Siberia. ACDV from three geographically distant localities segregated along geographic-specific lineages. Phylogenetic analysis based on complete S- and M-segment sequences showed that the new hantavirus had a common evolutionary origin with BRGV, harbored by the phylogenetically related European mole. The very close virus–host associations apparent through phylogenetic analyses of ACDV and BRGV, as well as for many other hantaviruses, are suggestive of co-evolution. On the other hand, mole-borne hantaviruses have been found in each of the four hantavirus genera, with evidence of host switching [9,19,20,22,33]. In other words, mole-borne hantaviruses appear to be more catholic, or eclectic, in their host proclivity than rodent-borne hantaviruses, suggesting that ancestral moles may have served as the early hosts of primordial hantaviruses [1].

The high prevalence of ACDV infection detected in Siberian moles suggests efficient transmission between individuals. Most probably, moles have a high risk of virus exposure due to spending their entire life underground. Hantaviruses are known to survive for prolonged periods in external environments [34]. Conceivably, virus in excretions from infected moles is protected from ultraviolet sunlight inactivation and is efficiently transmitted. High prevalence of ACDV in Siberian moles is consistent with the similarly high positivity rate of NVAV in European moles [17,23].

The European mole is known to serve as the reservoir host of more than one hantavirus species, NVAV and BRGV [17]. By contrast to NVAV, only 4.6% *Talpa europaea* were BRGV positive. We detected only ACDV among Siberian moles, possibly because only a limited number of specimens were tested. So, we are unable to conclude that NVAV or other NVAV-like hantaviruses are not hosted by Siberian moles.

The Siberian mole has a broad geographic range in the central part of Eurasia, spanning throughout the taiga zone of south-central Siberia in Russia, as far south as northern Mongolia and Kazakhstan. The high prevalence of ACDV in all three studied sites suggests that ACDV might be widespread throughout the distribution of *Talpa altaica*. Closely related virus and cyt B sequences of their hosts were detected in the site Academgorodok, located on the plains, while divergent virus strains and host mtDNA lineages were identified in the mountain site of Teletskoye. As previously shown, range expansions generally lead to a loss of genetic diversity along the expansion axis [35]. The low cyt B haplotype diversity, found in the northern plain territory (Academgorodok), might suggest rapid post glacial demographic expansion of *Talpa altaica* from the southern refugia in the Altai Mountains (Teletskoye) [36]. Site Azhendarovo is located close to the foothills of the Kuznetsk Alatau Mountains, in the contact zone of taiga and steppe with a fragmented landscape. Thus, two mtDNA lineages might originate from different local populations.

## 5. Conclusions

ACDV represents the seventh mole-borne hantavirus species, thus further expanding the host range diversity. Intensive studies are warranted to search for genetically distinct hantaviruses in other *Talpa* species, as well as in other New and Old World members of the Talpidae family.

## Figures and Tables

**Table 1:** Prevalence of hantavirus RNA and hantavirus sequences in *Talpa altaica* from Siberia, Russia.

		Number Hantavirus Positive/Tested		GenBank No.		
Capture Site	Year	Moles	Virus Strain	S	M	L
Novosibirsk Oblast, Academgorodok	2017	1/2	Academ-Ta450	MK340905	OL871119	MH784614
	2019	3/3	Academ-Ta348	-	-	MZ062416
			Academ-Ta400	-	-	MZ062417
			Academ-Ta589	-	-	MZ062418
	2021	2/2	Academ-Ta68	-	-	OL871122
			Academ-Ta101	-	-	OL871123
Altai Republic, Teletskoye	2018	1/1	Telet-Ta78			MZ062419
	2020	5/6	Telet-Ta601	MZ062425	-	MZ062420
			Telet-Ta602	-	-	MZ062421
			Telet-Ta603	MZ062426	-	MZ062422
			Telet-Ta604	-	-	MZ062423
			Telet-Ta615	MZ062427	-	MZ062424
Kemerovo Oblast, Azhendarovo	2021	2/4	Azhen-Ta261	OL871120	-	OL871124
			Azhen-Ta322	OL871121	-	OL871125

“-” sequences unavailable.

## Data Availability

GenBank accession numbers for sequence data are available in Table 1 and in Figure 2 and Figure 3. Other study data are available on request from the corresponding authors.

## Supplementary Materials

The following supporting information can be downloaded at: https://www.mdpi.com/article/10.3390/v14020309/s1, Table S1: Oligonucleotide primers for amplification of the S- and M-segments of soricine mole-borne hantaviruses.
