# Supplementary material for: Academ Virus, a Novel Hantavirus in the Siberian Mole (Talpa altaica) from Russia
Source: Viruses. 2022 Feb 2;14(2):309. doi: 10.3390/v14020309 (PMC8877406; doi:10.3390/v14020309)
Supplement: Supplementary file 1 [file viruses-14-00309-s001.zip › viruses-1559513-supplementary.pdf]

Table S1. Oligonucleotide primers used to amplify the S and M segments of mole-borne hantaviruses.

Segment	Primer	Sequence (5`-3`) (Polarity)
SML	**OSM55**	TAG TAG TAG ACT CC (+/-)
S	S2FR	TAG TAG TAK RCT CCC TAA ARA G (+/-)
	S437F	SWG GTC ARA CTG CHR AYT GG (+)
	S593R	GAC TGG GCA TTN GGC ATN GA (-)
	S2	AGC TCA GGA TCC ATG TCA TC (-)
	S952F	TGG GTN TTT GCW GGN GCA CC (+)
M	M-AC	CTG CAG TAG TAG TAK RCT CCG CAG (+/-)
	MC600R	TCA GGT TGR AAG CAT CTW CCY TC (-)
	M1200R	CCW CCC TCT GAR TAT GCC TCA CAT (-)
	MC790F	TGT RTW GCA GGW GGA AAT TCA GA (+)
	MC1020F	GTA TGT WTT YTC GAA RGA TGA AGA (+)
	MC1010R	TCT TCA TCY TTY GAR AAW ACA TAC AA (-)
	MA1490F	TGT GTN CCW GGN TTY CAT GG (+)
	M1685F	ACN AAG GGY TCW ATG GTN TGT GA (+)
	MC1710R	CAC TCT AAT TTG CAR AYA TCA CAW ACC (-)
	M2560F	GGW ACT GTT TCW AAA TTT CAR CCW GGA G (+)
	M2631R	CAT GAT RTC NCC AGG RTC NCC (-)
	MC3100F	ATT CAG RGG AAA TTG GAT GG (+)

Abbreviations: A, Adenine; C, Cytosine; G, Guanine; H, A or C or T; K, G or T; N, any nucleotide; R, A or G; S, G or C; T, Thymine; W, A or T; Y, C or T.
